# Fear extinction rescuing effects of dopamine and L-DOPA in the ventromedial prefrontal cortex

**DOI:** 10.1038/s41398-023-02708-8

**Published:** 2024-01-08

**Authors:** Simone B. Sartori, Thomas M. V. Keil, Kai K. Kummer, Conor P. Murphy, Ozge Gunduz-Cinar, Michaela Kress, Karl Ebner, Andrew Holmes, Nicolas Singewald

**Affiliations:** 1 Department of Pharmacology and Toxicology, Institute of Pharmacy and Center for Molecular Biosciences Innsbruck, University of Innsbruck, Innsbruck, Austria; 2 Institute of Physiology, Department of Physiology and Medical Physics, Medical University of Innsbruck, Innsbruck, Austria; 3 Laboratory of Behavioral and Genomic Neuroscience, NIH/NIAAA, Rockville, MD USA

**Keywords:** Psychiatric disorders, Learning and memory

## Abstract

The ventromedial prefrontal cortex (vmPFC; rodent infralimbic cortex (IL)), is posited to be an important locus of fear extinction-facilitating effects of the dopamine (DA) bio-precursor, L-DOPA, but this hypothesis remains to be formally tested. Here, in a model of impaired fear extinction (the 129S1/SvImJ inbred mouse strain; S1), we monitored extracellular DA dynamics via in vivo microdialysis in IL during fear extinction and following L-DOPA administration. Systemic L-DOPA caused sustained elevation of extracellular DA levels in IL and increased neuronal activation in a subpopulation of IL neurons. Systemic L-DOPA enabled extinction learning and promoted extinction retention at one but not ten days after training. Conversely, direct microinfusion of DA into IL produced long-term fear extinction (an effect that was insensitive to ɑ-/ß-adrenoreceptor antagonism). However, intra-IL delivery of a D1-like or D2 receptor agonist did not facilitate extinction. Using ex vivo multi-electrode array IL neuronal recordings, along with ex vivo quantification of immediate early genes and DA receptor signalling markers in mPFC, we found evidence of reduced DA-evoked mPFC network responses in S1 as compared with extinction-competent C57BL/6J mice that were partially driven by D1 receptor activation. Together, our data demonstrate that locally increasing DA in IL is sufficient to produce lasting rescue of impaired extinction. The finding that systemic L-DOPA increased IL DA levels, but had only transient effects on extinction, suggests L-DOPA failed to reach a threshold level of IL DA or produced opposing behavioural effects in other brain regions. Collectively, our findings provide further insight into the neural basis of the extinction-promoting effects of DA and L-DOPA in a clinically relevant animal model, with possible implications for therapeutically targeting the DA system in anxiety and trauma-related disorders.

## Introduction

Cognitive behavioural therapy involving exposure to fear eliciting cues is an effective treatment for anxiety and trauma-related disorders [1–3]. During an extinction session, repeated exposure to a cue (conditioned stimulus, CS) that predicts a previously experienced aversive outcome (unconditioned stimulus, US) produces a reduction in conditioned responses via formation of a CS-no US association that behaviourally competes with the original CS-US memory. Deficits in fear extinction are reported in diverse anxiety- and trauma-associated disorders [e.g. post-traumatic stress disorder (PTSD)], taking the form of impaired extinction learning and/or subsequent cue- or context-driven fear relapse ([4, 5], for review see [6]). Hence, identifying the key loci within the neural circuitry underlying extinction deficits could inform strategies for the augmentation of fear extinction as a treatment for these disorders (for review see [7, 8]).

In recent years, we and other research groups have demonstrated a role of the neurotransmitter dopamine (DA) in fear extinction (for recent reviews see [9–11]) as enhancing DA levels by systemic administration of the DA bio-precursor, L-DOPA, DA or the non-selective monoaminergic drug, methylphenidate, facilitates extinction memory consolidation in healthy humans [12–15] and extinction-competent rodents, such as the C57BL/6 J (BL6) mouse strain [12, 16–18]. However, the neural loci of L-DOPA’s effects on extinction are only now beginning to be unravelled [13, 15, 19, 20].

Previous studies have shown that loss of DAergic neurons by intra-mPFC injection of 6-OHDA delays extinction learning and impairs retrieval [21, 22], whereas facilitation of extinction by L-DOPA is accompanied by increased activation in human vmPFC and mouse infralimbic cortex (IL; also area 25, consistent with the human subgenual anterior cingulate cortex [23–25]) [12]. Moreover, pharmacological blockade of D2 and D1 receptors specifically within IL has been shown to impair fear extinction learning and retrieval [26–29]. These observations are particularly notable because the vmPFC and IL—though the exact human analogous subregion of the vmPFC needs to be still clarified [25]—are strongly implicated in fear extinction ([30–33]; for review see [6, 34–37]): for example, vmPFC/IL recruitment during early extinction predicts successful extinction consolidation [30, 38–42] and the efficacy of therapeutic interventions [36, 43].

In this context, systemic administration of L-DOPA increases vmPFC activity during extinction consolidation in healthy volunteers in a manner predicting the later strength of extinction memory [13]. In addition, long-term rescue of deficient fear extinction in the 129S1/SvImJ (S1) mouse strain following a dietary, multimodal Zinc-targeting intervention is associated with normalization of aberrant neuronal activity and upregulation of DA receptor genes in mPFC [20, 44]. However, L-DOPA’s ability to rescue fear extinction in S1 mice over time periods longer than 24 h is limited [20]. Along similar lines, a recent study found that L-DOPA’s extinction-facilitating effects and associated vmPFC (re-)activation are attenuated in women with PTSD [19]. Hence, while these findings suggest the vmPFC/IL is a key locus of the fear extinction-promoting effects of L-DOPA [12, 13, 20], the drug’s actions in this region and its associated effects on extinction could be limited in individuals with deficient extinction.

Recent work has shown that, during fear extinction, DA neurons in the ventral tegmental area (VTA) signal a positive prediction error on omission of the expected aversive outcome (footshock) that drives extinction [45–47] and safety learning [48]. Indeed, DA levels rise during fear extinction training in the medial prefrontal cortex (mPFC) [49, 50]. However, since VTA DA projections to the mPFC are sparse [51] and seem to be involved in the encoding of aversive events [52–54] and in fear extinction impairment, rather than facilitation [45], extinction-related mPFC DA has been suggested to originate from another source, for example locus coeruleus noradrenergic neurons [55].

To address these questions in the current study, we used the S1 strain characterised by insufficient fear extinction—a hallmark of anxiety—and trauma-related disorders including PTSD, panic disorder and generalised anxiety disorder. S1 mice display several other clinical features including deficient safety learning, overgeneralization of fear, lower heart rate variability and, at the neural level, dysfunctions in key brain areas of the extinction neurocircuitry including the mPFC and amygdala, consistent with imaging studies of patients with PTSD [for review see [56]]. We examined the extinction-related effects of L-DOPA and DA in the S1 mice, and combined these behavioural analyses with in vivo measurement of DA (microdialysis) and ex vivo analysis (immediate early gene (IEG) activation, gene expression) of DA-related activity in mPFC/IL. Our findings provide further evidence that IL is a key locus of DAergic facilitation of extinction and show that systemic L-DOPA extinction-promoting effects are attenuated in animals with deficient extinction.

## Materials and methods

### Animals

All experimental procedures on adult male S1 and BL6 mice were approved by the national ethical committee on animal care and use (Bundesministerium für Bildung, Wissenschaft und Forschung) in compliance with international laws and policies (for further details on this and all other aspects of the Materials and methods, see Supplemental Information).

### Stereotaxic surgery

Guide cannulae for microinfusers were implanted above the IL target according to previous protocols [57], focusing on the rostral part of IL because of its strong connections to the basal amygdala, relative to more caudal IL [58]. Microdialysis probes were too large to be restricted exclusively to IL and therefore targeted a larger region encompassing IL and dorsally-neighbouring prelimbic cortex (PL) which we refer to collectively as mPFC. During recovery, single-housed mice received analgesic care.

### Extinction of cued conditioned fear

Animals were cue fear conditioned by using three pairings of a tone cue (75 dB, 30 s; CS) with a co-terminating mild, scrambled foot shock (0.6 mA, 2 s; US) in context A. Fear extinction was performed by exposing animals to 16 CS for training and two CS tones for retrieval in a novel context B which differed from context A in visual, olfactory and spatial signals on experimental days 2, 3 and 13, respectively, (ABBB design) according to previous protocols [20, 57].

### In vivo microdialysis measurements of extracellular mPFC DA

**Fig. 1:**
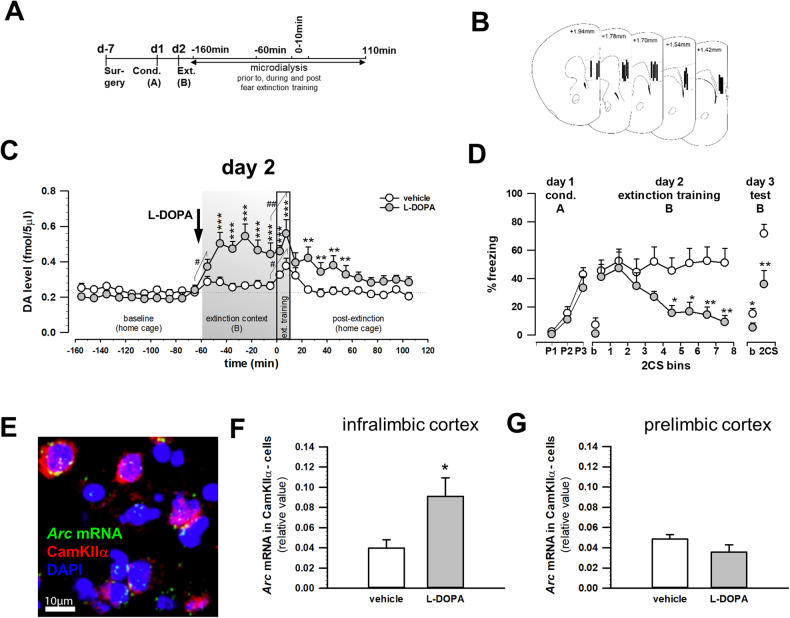
L-DOPA augments extinction-related mPFC DA levels and neuronal activity. **A** Experimental schematic diagram of in vivo microdialysis experiments to measure extracellular DA levels in mPFC of S1 mice following systemic administration of L-DOPA (20 mg/kg i.p.) one hour before fear extinction training. **B** Placement of the microdialysis probes in mPFC. **C** Dynamic changes in extracellular DA levels in mPFC of S1 mice during fear extinction training following systemic administration of L-DOPA (*n* = 9) or vehicle (*n* = 8). Data points represent DA concentrations of 10 min microdialysates with the exception of two 5 min fractions (each covering eight CSs) collected during fear extinction training. Grey boxes indicate the time period when animals were exposed to the extinction context B and to 16 non-reinforced CSs for extinction training on experimental day 2. **D** Systemic L-DOPA (*n* = 8) prior training generated extinction in fear conditioned (three CS-US pairings, P1-P3) S1 mice as indicated by lower levels of CS-related freezing on extinction training and drug-free extinction retrieval one day later as compared with vehicle treatment (*n* = 9). Bins of two CSs are shown. Freezing levels displayed by animals prior to first CS presentation (baseline, b) were low. **E** High magnification representative image depicting *Arc* mRNA expression in CaMKIIα-positive (red cytoplasmic staining around the nuclear DAPI counterstain) and CaMKIIα-negative cells in IL. **F**, **G** Fear extinction training induced *Arc* mRNA in IL and PL. The number of cells expressing *Arc* mRNA (expressed relative to the number of DAPI-stained cells) was increased in CaMKIIα-negative cells in IL and not PL of L-DOPA-treated S1 mice (*n* = 7) as compared with vehicle treatment (*n* = 7). Data are mean ± sem. *p < 0.05, **p < 0.01, ***p < 0.001 L-DOPA vs. vehicle, ^#^*p* < 0.05, ^##^*p* < 0.01 within treatment effect analysed by a Fisher’s LSD test following a significant two-way ANOVA with repeated measures and a two-tailed *t*-test, respectively. A: context A; b: baseline; B: context B; CamKIIα-: CamKIIα-negative; cond.: cued fear conditioning; CS: conditioned stimulus; d: day; ext.: fear extinction training; P1-3: CS-US pairing.

Microdialysis fractions of 5 and 10 minutes, respectively, were continuously collected in rostral mPFC of freely moving mice, as previously described [59], before, during and after fear extinction sessions (Fig. 1A). DA concentrations in microdialysates were determined by using high-performance liquid chromatography with electrochemical detection according to our published protocol [59].

### Systemic and intra-IL drug administration

For systemic administration, L-DOPA (20 mg/kg), benserazide (12.5 mg/kg), the D1-like receptor agonist SKF-81297 (10 mg/kg) and the selective D2 receptor agonist sumanirole (10 mg/kg) were dissolved in saline or saline containing 1% DMSO and 3.3% Tween 80 for systemic (i.p., 10 ml/kg) administration. For intra-IL administration, DA (40 µg/hemisphere), SKF-81297 (0.1 µg/hemisphere), sumanirole (10 µg/hemisphere), phentolamine (20 µg/hemisphere) and timolol (20 µg/hemisphere) were dissolved in artificial cerebrospinal fluid (aCSF).

### Multi-electrode array (MEA) mPFC neuronal recordings

Spontaneous action potential discharge activity was recorded in acute mPFC coronal brain slices (300 µm) according to a previous protocol [60] under physiological conditions and in the presence of DAergic ligands.

### Quantitative polymerase chain reaction (qPCR)

Reverse qPCR with forward and reverse primers designed against the target of interest was performed on complementary cDNA derived from homogenised frozen tissue punches containing both IL and PL subregions using a commercially available kit (Qiagen Biosciences, USA) in combination with Fast SYBR green master mix (Applied Biosciences, USA).

### Quantitative D1-like and D2-like receptor autoradiography

D1-like and D2-like receptors were visualized on frozen tissue sections (12 µm) using [^3^H]-SCH-23390 (1 nM) and [^3^H]-nemonapride (1 nM) according to previous protocols [61]. Receptor density was determined in IL and PL, which were identified with reference to a mouse brain atlas, from digitized autoradiograms.

### Arc fluorescence in situ hybridization (FISH) in mPFC neurons

mRNA expression of the activity-regulated cytoskeleton-associated protein (*Arc*) was detected by TSA-amplified FISH using a customized, fluorescein-labelled probe (Exiqon, Denmark) according to a previous protocol [62] and quantified in IL and PL by fluorescent microscopy.

### IEG immunohistochemistry of pERK and pCREB in mPFC neurons

Immunohistochemistry was performed on free-floating brain slices (40 µm) using rabbit anti-pCREB and rabbit anti-pERK (Cell Signalling Technology, USA) as primary antibodies and a biotinylated goat anti-rabbit secondary antibody (Vector Laboratories, USA). The number of immuno-positive cells was quantified in IL and PL.

### Statistical analysis

Data are expressed as mean ± standard error of the mean (sem). Statistical analysis of data was performed using Statistica 13 (StatSoft Europe). The level of statistical significance was set to *p* < 0.05.

## Results

### L-DOPA rescues fear extinction and augments extinction-related mPFC DA levels

We first performed in vivo microdialysis to monitor extracellular DA concentrations in mPFC (Fig. 1A–C) of S1 mice during fear extinction training after systemic treatment with a dose of L-DOPA-previously shown to rescue fear extinction in this mouse strain [20]. On experimental day 1, mice were fear conditioned to a tone CS in context A [pairing effect: F(2,34) = 37.41, *p* < 0.001; Fig. 1D]. On the next day microdialysates were collected in the mPFC of both experimental groups in home cages until stabilized at an average of 0.22 ± 0.01 fmol/sample within the first 100 minutes [time: F(9,135) = 1.30, *p* = 0.243; time × treatment: F(9,135) = 1.25, *p* = 0.273; Fig. 1C] reflecting baseline. Then, either L-DOPA (20 mg/kg i.p.) or vehicle was administered i.p. one hour before fear extinction training and mice were placed into context B (differing from context A in visual, olfactory and spatial cues) for microdialysate collection during a further one-hour baseline period followed by extinction training.

In mPFC dynamic changes in extracellular DA concentrations were observed on the day of extinction training [time × treatment: F(27,378) = 4.91, *p* < 0.001; Fig. 1C]. L-DOPA produced a pronounced and sustained increase in extracellular DA levels, as compared to vehicle, during the context B baseline period. Importantly, extinction training was associated with a further increase in DA levels over context B baseline, to the same extent in both treatment groups, but at an overall higher level in L-DOPA-treated mice than vehicle-treated controls (Fig. 1C). In L-DOPA-treated animals DA levels remained higher relative to vehicle-treated controls for another 50 minutes and above baseline throughout the whole observation period after extinction training (Fig. 1C).

Behaviourally, replicating previous data [20], L-DOPA promoted fear extinction learning in S1 mice [time x treatment interaction: F(7,91) = 4.14, *p* < 0.001; Fig. 1D] and the formation of a fear extinction memory—as indicated by lower levels of CS-related freezing during extinction training and drug-free retrieval testing the following day as compared with vehicle controls. On extinction retrieval test, however, freezing levels of both L-DOPA and vehicle-treated groups was higher than at the end of the extinction training and is most likely to reflect fear incubation, a phenomenon that has been reported previously in the extinction-impaired S1 strain [63].

These data demonstrate that the extinction-rescuing effect of L-DOPA is associated with an increase in mPFC DA levels in vivo.

### L-DOPA alters extinction-related neuronal activity in mPFC

To complement these findings, we examined the effects of systemic L-DOPA on extinction-related neuronal activity in mPFC via expression of the IEG Arc—which has been previously shown to be upregulated following successful fear extinction training [64, 65]. In S1 mice we found that L-DOPA treatment did not affect overall *Arc* mRNA expression in IL following extinction training [t(12) = 0.176, *p* = 0.863; data not shown, see also Fig. S1C). However, when we separately analysed *Arc* mRNA expression in cells co-labelled with calcium/calmodulin-dependent protein kinase II alpha (CaMKIIα; Fig. 1E), a marker primarily, though not solely (e.g., [66, 67]), for excitatory pyramidal neurons [68], we observed an increase in *Arc* mRNA expression exclusively to CaMKIIα-negative cells in the IL following L-DOPA treatment [t(12) = 2.560, *p* = 0.025; Fig. 1F]. This effect appeared to be most pronounced for cells expressing *Arc* mRNA in both cytoplasm and nucleus of CaMKIIα-negative cells (Fig. S1E) marking neurons persistently active throughout training as revealed by cellular compartment FISH (catFISH) analysis. Lastly, we observed no L-DOPA-related effects on *Arc* labelling in PL [overall: t(12) = 0.655, *p* = 0.525; CaMKIIα-negative cells: t(12) = 1. 477, *p* = 0.165; Fig. 1F; see also Fig. S1D–F].

These data indicate that systemic L-DOPA treatment produces an extinction-related increase in neuronal activity in a specific subset of IL, but not PL, neurons.

### Direct infusion of DA into IL rescues deficient extinction

**Fig. 2:**
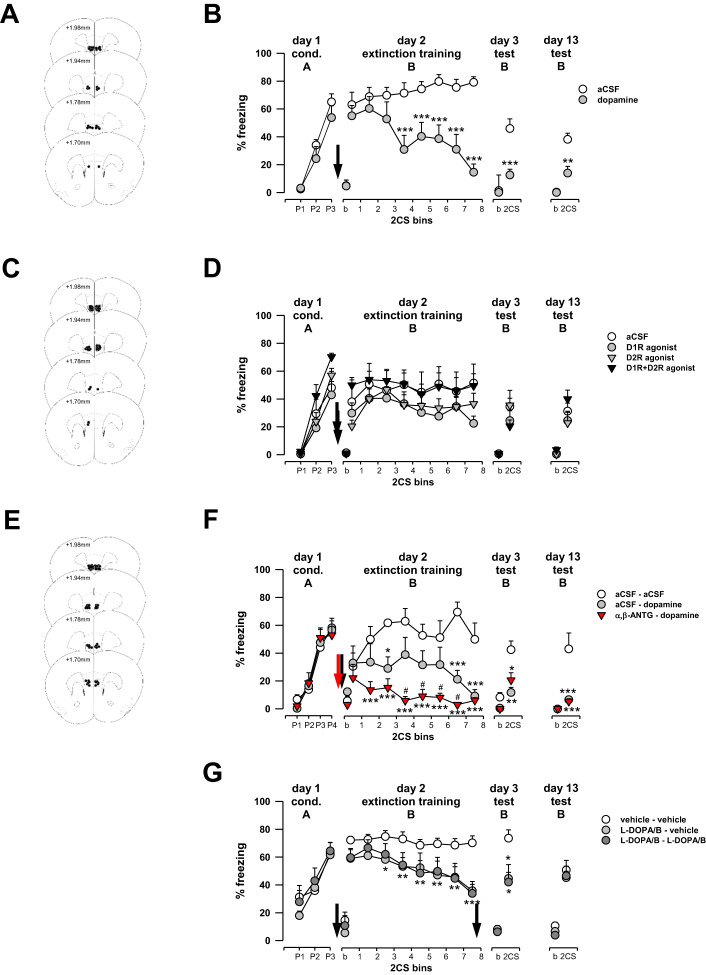
Direct infusion of DA into IL rescues deficient extinction. Fear conditioned (three CS-US pairings, P1-P3) S1 mice were subjected to fear extinction training and two test sessions on experimental days 2, 3 and 13. Bins of two CSs are shown. Freezing levels displayed by animals prior to first CS presentation (baseline, b) were low. **A**, **C**, **E** Placement of microinfusion probes in IL. **B** Pre-extinction training application of DA (*n* = 9) into IL of S1 mice [pairing: F(2,34) = 56.89, *p* < 0.001; pairing × treatment: F(2,34) = 0.76, *p* = 0.476] decreased CS-elicited freezing levels during extinction training and extinction retrieval tests performed on experimental days 3 and 13 as compared with vehicle treatment (*n* = 10). **D** Microinfusion of the selective D1-like receptor agonist SKF-81297 (*n* = 10), the D2 receptor agonist sumanirole (*n* = 11) or the combination of the two (*n* = 7) into IL prior to fear extinction training did not affect CS-related freezing levels in S1 mice during extinction training or extinction retrieval tests performed on experimental day 3 and day 13 as compared with vehicle (*n* = 10). **F** Intra-IL infusion of the α,β-adrenoceptor blockers, timolol and phentolamine, prior to intra-IL DA infusion (*n* = 10) and subsequent extinction training reduced freezing levels during later extinction trial-blocks to a greater extent than DA alone. Freezing levels on extinction retrieval tests performed on day 3 and day 13 were similarly lower in the DA-alone (*n* = 10) and drug-combination groups (*n* = 10) as compared with vehicle (*n* = 5). **G** Administration of combined L-DOPA and benserazide either only prior (*n* = 10) to or prior to and post (*n* = 10) extinction training produced a similar decrease in freezing levels during extinction training and extinction retrieval test performed on experimental day 3 and 13 as compared with vehicle (*n* = 10). Arrows (DAergic drugs in black, α,β-adrenoceptors in red) denote the time point of the local or systemic administration of drug or vehicle. Data are presented as mean ± sem. *p < 0.05, **p < 0.01, ***p < 0.001 DA-treated groups vs. vehicle (aCSF), ^#^*p* < 0.05 α,β-adrenoceptor antagonists-DA-treated group vs. aCSF-DA treated group as analysed by a one- or two-way ANOVA followed by Fisher’s LSD test. A: context A; aCSF: artificial cerebrospinal fluid; ANTG: antagonist; b: baseline; B: context B; cond.: cued fear conditioning; D1R: dopamine D1 receptor; D2R: dopamine D2 receptor; L-DOPA/B: combined administration of L-DOPA with benserazide; P1-3: CS-US pairing; test: extinction retrieval test.

Our microdialysis and neuronal activation data indicate a concordance between the effects of systemic L-DOPA on mPFC DA levels, IL neuronal activation and a rescue of extinction in otherwise impaired animals. Given this link, we next asked whether direct administration of DA into IL per se was sufficient to recapitulate the effects of systemic L-DOPA. In fear-conditioned S1 mice [pairing: F(2,34) = 56.89, *p* < 0.001; pairing x treatment: F(2,34) = 0.758, *p* = 0.476] intra-IL DA microinfusion (Fig. 2A) prior to extinction training caused decreased freezing levels, relative to vehicle infusion, on late not early, extinction training CS trial-blocks [CS × treatment: F(7,119) = 6.93, *p* < 0.001; Fig. 2B]. Intra-IL DA-treated animals continued to show lower freezing levels on drug-free extinction retrieval tests, both at one [t(17) = 4.00; *p* < 0.001] and ten [t(15) = 3.63; *p* = 0.003) days after extinction training as compared with vehicle-treated controls (Fig. 2B).

Previous studies have implicated D1 and D2 receptors, including those expressed in IL, in fear extinction [26–29, 69, 70]. This led us test whether the pro-extinction effects of intra-IL DA (Fig. 2B) could be mimicked by activation of D1 or D2 receptors in IL of fear-conditioned S1 mice [pairing: F(2,74) = 87.65, *p* < 0.001; pairing x treatment: F(6,74) = 1.72, *p* = 0.129]. Pre-training intra-IL infusion (Fig. 2C) of either a D1-like receptor agonist (SKF-81297) or a D2 receptor agonist (sumanirole), or a combination of the two drugs did not affect, relative to vehicle, freezing levels during extinction training [CS × treatment: F(21,259) = 1.00, *p* = 0.461] and subsequent one day or ten day post-training extinction retrieval tests [day 3: F(3,37) = 0.84, *p* = 0.480] and day 13 [F(3,36) = 1.37, *p* = 0.266; Fig. 2D).

These findings demonstrate that intra-IL delivery of DA, but not D1-like or D2 receptor activation alone, is sufficient to produce long-lasting rescue of impaired extinction.

### Adrenoceptor blockade enhances intra-IL DA rescue of deficient extinction

DA can also bind to adrenergic receptors, though with lower affinity than to its preferred DA receptors and, additionally, can be converted to noradrenaline (NA) [71]. Since mPFC NA has been shown to alter fear extinction via α- and β- adrenoreceptors ([72], for review see [73, 74]), it may be speculated that NA contributed to the DA-related rescue of extinction we observed in S1 mice. To address this possibility, we microinfused a mixture of the adrenoceptor blockers, pan-α- (phentolamine) and pan-β (timolol), into IL of fear conditioned S1 mice [pairing: F(3,66) = 61.49, *p* < 0.001; pairing × treatment: F(6,66) = 0.344, *p* = 0.911] ten minutes prior to intra-IL DA infusion (Fig. 2E), and then tested for extinction training.

We found—replicating our earlier result—that intra-IL DA reduced freezing levels on late extinction training trial-blocks, relative to vehicle controls. In addition, we observed that pre-treatment with the two adrenoceptor blockers accelerated the extinction-related decrease in freezing levels (i.e., effect evident on intermediate trial-blocks), relative to DA-alone [trial × treatment: F(14,112) = 3.24, *p* < 0.001; Fig. 2F). Freezing levels were lower in the DA+adrenoceptor blocker and DA-alone groups than vehicle controls on one-day and ten-day extinction retrieval tests, but the treatment groups did not differ from one another [day 3: F(2,21) = 7.32, *p* = 0.004 and day 13 [F(2,19) = 19.39, *p* < 0.001; Fig. 2F].

The effects of these adrenoceptor blockers suggest that NA activation of adrenoceptors opposes the extinction rescuing effects of intra-IL administration of DA. Though this effect was only evident as lower freezing in the adrenoceptor blocker group during extinction learning, it may have been occluded by low ‘floor’ levels of freezing during extinction retrieval.

### Augmenting L-DOPA-induced DA does not prevent long-term fear relapse

Thus far, our results show that increasing IL DA levels is sufficient to rescue extinction learning and retrieval for up to ten days post-training. However, while we showed here that systemic L-DOPA rescued extinction retrieval one day post-training, we had previously shown that this effect did not persist at the ten-day retrieval timepoint [20]. The transient nature of the L-DOPA effect is of potential clinical importance given the efficacy of L-DOPA as an adjunct for extinction-based therapies assumes its ability to confer long-term protection against fear relapse [19].

This led us to ask whether a second dose of systemic L-DOPA would produce sustained DA increases into the extinction consolidation period and longer-lasting effects on extinction. L-DOPA was administered to fear-conditioned S1 mice [pairing: F(2,54) = 40.18, *p* < 0.001; pairing × treatment: F(4,54) = 0.674, *p* = 0.613] either prior to extinction training or both prior to and immediately after extinction training. In this experiment, L-DOPA was co-administered with the decarboxylase inhibitor benserazide as a means to prevent peripheral decarboxylation of L-DOPA to DA [75]. We found that, relative to vehicle-treated controls, systemic L-DOPA administration decreased freezing levels on late extinction training trial-blocks [CS × treatment: F(7,196) = 2.07, *p* = 0.049] and drug-free extinction retrieval testing the next day [F(2,17) = 3.83, *p* = 0.042), but not on the ten-day test [F(2,19) = 0.20, *p* = 0.822, Fig. 2G], irrespective of whether L-DOPA was administered once or twice.

The findings of this experiment replicate the short-term extinction-promoting effect of L-DOPA in S1 mice and extend these data by showing that a second dose of L-DOPA during the extinction consolidation period did not bolster the long-term effects of L-DOPA on extinction.

### Abnormal DA-mediated mPFC neuronal responses in extinction-deficient mice

**Fig. 3:**
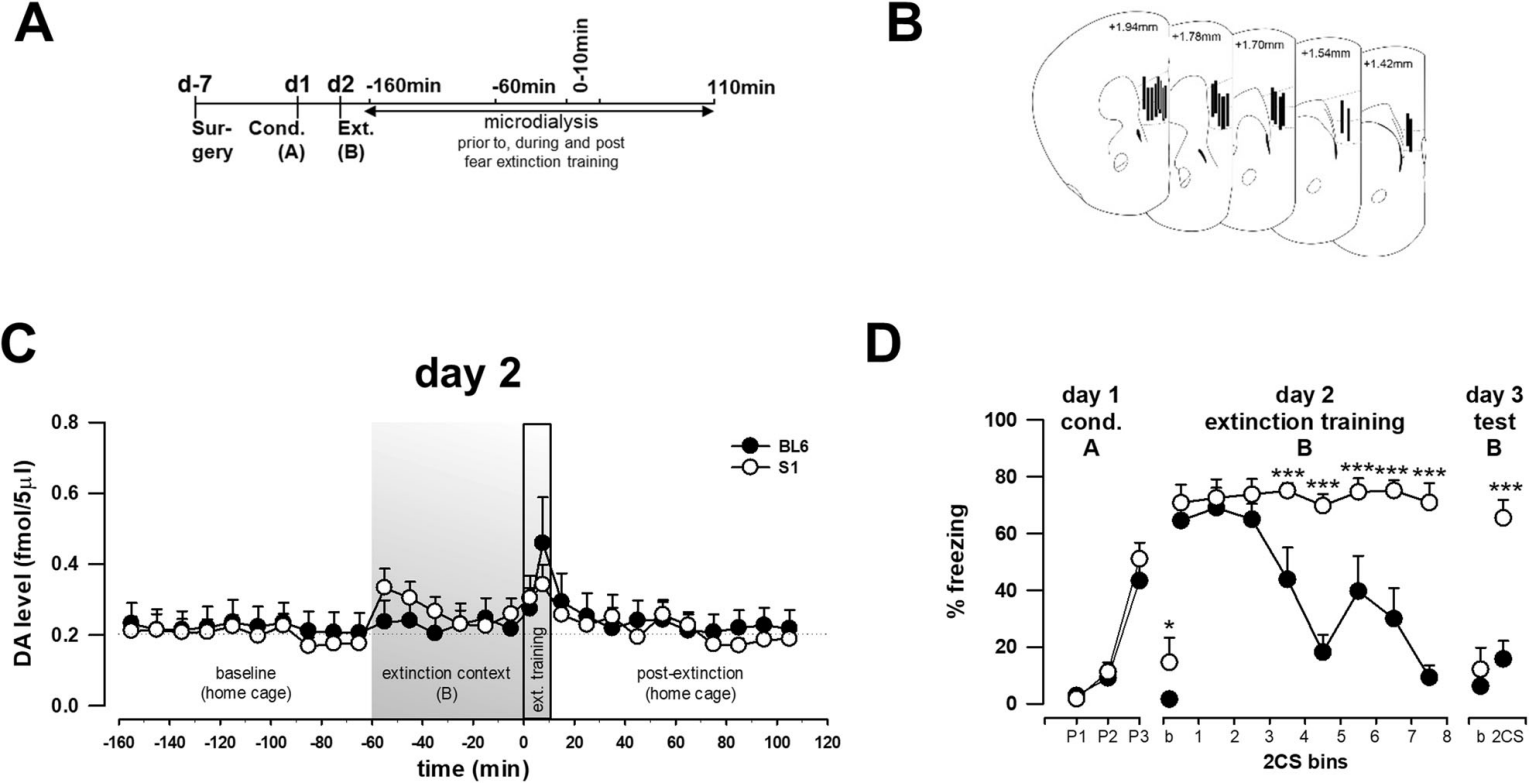
Comparison of mPFC DA levels during extinction in extinction deficient and intact animals. **A** Experimental design of in vivo microdialysis experiments for measuring extracellular mPFC DA levels in S1 and BL6 mice during fear extinction. **B** Placement of microdialysis probes in mPFC. **C** Dynamic changes in mPFC DA levels in S1 (*n* = 10) and BL6 (*n* = 9) mice during extinction training. Data points represent DA concentrations of 10 min microdialysates with the exception of two 5 min fractions (each covering eight CSs) collected during fear extinction training. Grey boxes indicate the time period when animals were exposed to the extinction context B and to 16 non-reinforced CSs for extinction training on experimental day 2. Date are mean ± sem. **D** Freezing levels decreased over extinction training and retrieval testing in fear conditioned (three CS-US pairings, P1-P3) BL6 (*n* = 8) but not S1 (*n* = 10) mice. Bins of two CSs are shown. Freezing levels displayed by animals prior to first CS presentation (baseline, b) were low. ***p < 0.001 S1 vs. BL6. Analysed by two-way ANOVA with repeated measures followed by Fisher’s LSD test. A: context A; b: baseline; B: context B; BL6: C57BL/6 J; cond.: fear conditioning: CS: conditioned stimulus; d: day; ext.: fear extinction training; P1-3: CS-US pairing.

The inability of L-DOPA treatment to protect against post-extinction long-term recovery of fear in S1 mice (Fig. 2G, [20]) could potentially reflect abnormal DAergic signalling in mPFC. To investigate this possibility, S1 and extinction-competent BL6 mice (for previous comparison of these behavioural phenotypes, see e.g., [44, 63]) were fear-conditioned [pairing: F(2,32) = 69.03, *p* < 0.001; pairing x strain: F(2,36) = 1.28, *p* = 0.289; Fig. 3D] and mPFC DA dynamics between the two strains were compared the next day using in vivo microdialysis (as described above; Fig. 3A, B). Similar, dynamic changes in extracellular DA concentrations were revealed in S1 and BL6 mice on the day of extinction training [time: F(27,432) = 4.44, *p* < 0.001; strain × time: F(27,432) = 1.09, *p* = 0.349; Fig. 3C]. After an acclimation period in the home cage, DA levels were stable at 0.22 ± 0.02 fmol/sample in BL6 and 0.20 ± 0.01 fmol/sample in S1 mice [time: F(9,144) = 1.505, *p* = 0.151] and did not differ between the strains [strain: F(1,16) = 0.106, *p* = 0.750], irrespective of time [strain × time: F(9,144) = 0.272, *p* = 0.981; Fig. 3C].

On transfer to the extinction context B, there was a greater initial increase in mPFC DA levels in S1 than BL6 mice – a response likely due to an effect of handling and/or exposure to the novel context, as this difference resolved prior to extinction training. During extinction training, the strains displayed a similar CS-related increase in DA levels (*p* < 0.09 + 2.5 min vs. −0.5 min and −15 min; *p* < 0.05 + 2.5 min vs. −25 min and all baseline timepoints) with a peak increase later in training (*p* < 0.001 + 7.5 min vs. all timepoints before). On return to the home cage, both strains showed a decrease to similar baseline DA levels within ~20 min. Despite the similarity of DA dynamics during extinction, S1 mice exhibited higher levels of CS-related freezing than BL6 mice on training [strain × time: F(7,98) = 11.43, *p* < 0.001] and extinction retrieval testing the next day [t(16) = 4.42, *p* < 0.001; Fig. 3D].

**Fig. 4:**
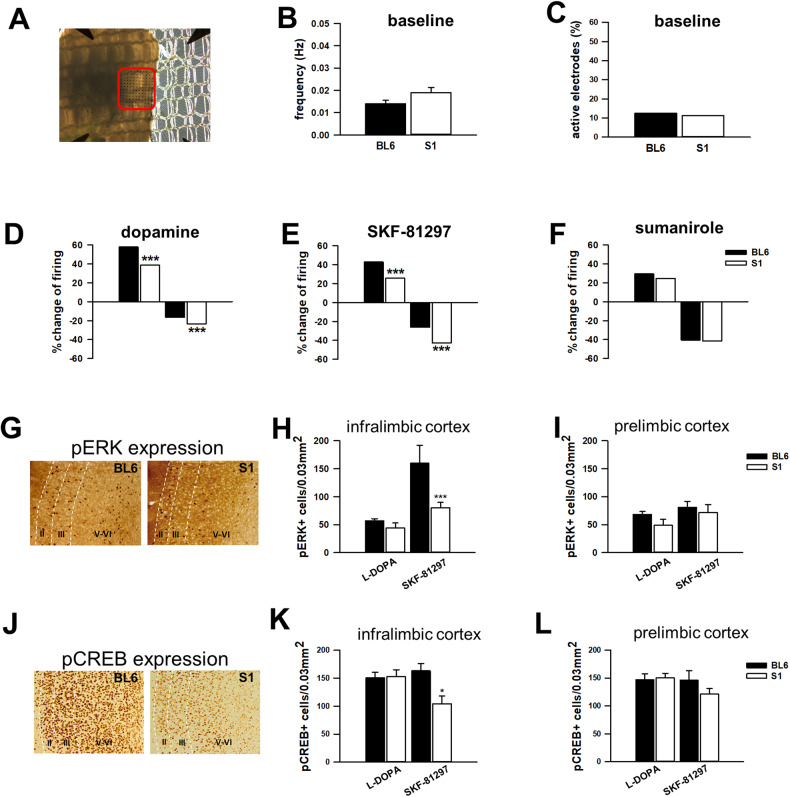
Abnormal DA-mediated mPFC neuronal responses in extinction-deficient mice. **A**–**C** Neuronal responses in the mPFC of S1 and BL6 mice as determined by ex vivo multi-electrode array (MEA) recordings. Example image showing mPFC mounted on the MEA probe. Spontaneous firing activity of mPFC neurons in S1 and BL6 mice, as indicated by averaged action potential firing frequency and the number of electrically active MEA sites. *n* = 4. **D**–**F** Bidirectional changes (increases and decreases) in spiking activity differed between S1 and BL6 mice in response to DA and the selective D1-like receptor agonist SKF-81297, but not to the D2 receptor agonist sumanirole. *n* = 4. **G**, **J** Representative images of pERK expression and pCREB expression after systemic administration of L-DOPA or the D1-like receptor agonist SKF-81297 in mPFC of S1 (*n* = 7 per treatment) and BL6 (*n* = 5 per treatment) mice. Cortical layers are delineated for detailed quantitative analysis which is summarised in Table 1. **H** The number of pERK-positive neurons per 0.03 mm^2^ was reduced in IL of S1 relative to BL6 mice following systemic administration of SKF-81297 (*n* = 5 per experimental group). **K** The number of pCREB-positive cells was lower in IL of S1 than BL6 mice after SKF-81297 administration. **I**, **L** No strain differences in pERK and pCREB expression in PL in response to DA receptor agonists. Data are presented as mean ± sem. *p < 0.05, ***p < 0.001 S1 vs. BL6 as analysed by two-way ANOVA followed by a Holm-Sidak pairwise multiple comparison procedure or a Fisher’s LSD test.

These data show that deficient extinction in S1 mice is not related to abnormal extracellular DA availability in mPFC. This suggests that either DA produces abnormal neuronal responses in S1 mPFC neurons or other brain regions and neurotransmitters, respectively, are responsible. Therefore, to provide a functional readout of DA-mediated effects in mPFC, we performed ex vivo multi-electrode array (MEA) recordings comparing DA-evoked mPFC neuronal responses in S1 and BL6 mice (Fig. 4A). There were no differences between strains in spontaneous mPFC neuronal activity, as measured by firing rate (*p* = 0.12; Fig. 4B) and the percentage of basally active recording electrodes (*p* = 0.57; Fig. 4C). In response to bath application of DA, however, S1 mice had a lesser percentage of electrodes exhibiting increased activity than BL6 mice, and a greater percentage showing decreased activity (χ² = 32.44, *p* < 0.001; Fig. 4D). A similar pattern of strain differences was evident in response to application of the D1-like receptor agonist SKF-81297 (100 µM; χ² = 32.93, *p* < 0.001; Fig. 4E), whereas D2 receptor agonist sumanirole (100 µM) failed to affect activity in either strain (100 µM; χ² = 2.28, *p* = 0.24; Fig. 4F).

These results show that S1 and BL6 mice exhibited similar extracellular mPFC DA levels during extinction, but differ in mPFC neuronal responses to DA and D1-like receptor activation in a manner suggestive of a bias towards neuronal inhibition over excitation in S1 mice.

### Abnormal DA-mediated mPFC signalling in extinction-deficient mice

We next assessed whether altered DA-mediated network responses in mPFC neurons in S1 mice reflected abnormal DA receptor signalling in IL [76]. Using immunohistochemistry, we measured IL neuronal expression of phosphorylated ERK (pERK; Fig. 4G) and CREB (pCREB; Fig. 4J)—two canonical downstream DA signalling molecules [77, 78]—following systemic administration of L-DOPA or SKF-82197. As mPFC network responses were unaltered following application of sumanirole, we did not include it in this experiment fulfilling the 3Rs (reduction). We observed fewer pERK-positive cells in IL, particularly in layers II-III, in S1 than BL6 mice following administration of SKF-82197, but not L-DOPA [strain: F(1,12) = 6.99, *p* = 0.021; strain × treatment: F(1,12) = 3.73, *p* = 0.077; Fig. 4H, Table 1). A similar trend was evident for pCREB expression by strain and treatment [strain: F(1,19) = 3.729, *p* = 0.069; strain × treatment: F(1,19) = 2.09, *p* = 0.051], and post-hoc analysis of pCREB expression revealed a significantly lower number of pCREB-positive cells in IL of S1 mice after SKF-81297 administration relative to BL6 mice (Fig. 4K, Table 1]. Notably, these differences in pERK and pCREB were specific to IL, with no differences apparent in PL (pERK: strain × treatment: F(1,12) = 0.260, *p* = 0.620; pCREB: strain × treatment: F(1,19) = 0.1.336, *p* = 0.262; Fig. 4I, L, Table 1), cingulate or motor cortex (Supplementary Table S1).

**Table 1:** mPFC layer-specific expression of pERK and pCREB in S1 and BL6 mice following systemic administration of dopaminergic drugs.

	L-DOPA		SKF-81297		statistics	
	BL6	S1	BL6	S1	strain × treatment	
Infralimbic cortex						
pERK (15 min)						
Layer II	16.8 ± 2.49	10.9 ± 0.97	85.5 ± 19.8	28.1 ± 5.86***	F(1,11) = 9.484	*p* = 0.011
Layer III	22.3 ± 2.10	16.3 ± 2.60	45.0 ± 6.58	18.1 ± 1.09***	F(1,11) = 9.509	*p* = 0.010
Layer V-VI	17.8 ± 1.36	17.3 ± 6.43	46.8 ± 3.33	34.1 ± 6.77	F(1,12) = 1.458	*p* = 0.249
pERK (1 h)						
Layer II	11.2 ± 1.35	8.92 ± 1.35	30.9 ± 5.08	27.4 ± 5.88	F(1,19) = 0.015	*p* = 0.903
Layer III	20.4 ± 1.86	19.1 ± 1.41	12.2 ± 1.29	12.9 ± 1.41	F(1,19) = 0.382	*p* = 0.543
Layer V-VI	23.0 ± 0.79	28.7 ± 2.47	12.3 ± 0.90	12.9 ± 2.26	F(1,20) = 1.325	*p* = 0.263
pCREB(1 h)						
Layer II	59.3 ± 3.60	64.6 ± 4.69	57.5 ± 4.93	38.0 ± 5.77*	F(1,20) = 4.658	*p* = 0.044
Layer III	49.0 ± 3.50	47.7 ± 3.58	56.1 ± 3.39	38.8 ± 3.87	F(1,19) = 3.591	*p* = 0.073
Layer V-VI	42.2 ± 3.77	40.4 ± 4.73	49.5 ± 5.62	27.5 ± 4.92	F(1,19) = 3.336	*p* = 0.084
Prelimbic cortex						
pERK (15 min)						
Layer II	27.5 ± 2.41	18.4 ± 3.54	26.0 ± 3.61	32.8 ± 6.53	F(1,12) = 3.406	*p* = 0.090
Layer III	18.0 ± 2.70	13.9 ± 5.11	16.4 ± 3.31	14.8 ± 2.72	F(1,11) = 0.506	*p* = 0.492
Layer V-VI	23.0 ± 1.86	16.5 ± 3.05	38.5 ± 4.58	24.4 ± 5.79	F(1,12) = 0.865	*p* = 0.371
pERK (1 h)						
Layer II	22.4 ± 0.74	23.4 ± 1.93	15.9 ± 2.00	17.1 ± 1.85	F(1,19) = 0.003	*p* = 0.958
Layer III	16.6 ± 0.86	19.9 ± 1.27	9.40 ± 1.41	13.0 ± 2.01	F(1,20) = 0.006	*p* = 0.938
Layer V-VI	20.9 ± 2.78	23.8 ± 0.75	16.6 ± 1.94	16.3 ± 1.75	F(1,19) = 0.617	*p* = 0.442
pCREB(1 h)						
Layer II	62.1 ± 3.52	64.1 ± 2.86	60.0 ± 5.28	49.4 ± 4.81	F(1,19) = 1.817	*p* = 0.194
Layer III	45.0 ± 3.31	48.6 ± 1.99	48.1 ± 3.50	43.9 ± 2.64	F(1,19) = 1.580	*p* = 0.224
Layer V-VI	39.9 ± 5.21	37.8 ± 3.93	38.0 ± 8.49	28.5 ± 3.18	F(1,19) = 0.456	*p* = 0.508

The expression of pERK and pCREB was investigated in mPFC subregions by a layer-specific analysis 15 min or 1 h after the administration of L-DOPA (20 mg/kg) and the selective D1-like receptor agonist SKF-81297 (10 mg/kg). The number of pERK- and pCREB-positive cells in a representative area of 0.01 mm^2^ per layer was counted. *n* = 4–7 per experimental group.

*p < 0.05, ***p < 0.001 S1 vs. BL6. Analysed by two-way ANOVA and a post-Fisher’s LSD test.

Finally, using FISH, we examined in S1 and BL6 mice the extinction-related mRNA expression of the IEG *Arc*, which is downstream of several converging signalling pathways including the D1 receptor-ERK/CREB transduction [76, 77, 79]. Relative to BL6 mice, *Arc* mRNA expression was reduced in IL [strain: F(1,42) = 6.260, *p* = 0.016, Fig. S1G], but not in PL [strain: F(1,41) = 1.565, *p* = 0.218, Fig. S1H].

These data show that D1-like receptor-mediated signalling is blunted in IL of S1 mice. This effect likely reflects a functional abnormality, rather than reduced D1 receptor expression, as we found no strain differences in basal IL gene expression or number of receptor binding sites for D1 or other DA receptor subtypes (Table 2).

**Table 2:** Expression of DA receptors in mPFC of naïve S1 and BL6 mice.

	BL6	S1	t	*p*
Gene expression in mPFC (relative expression)				
DRD1	1.0 ± 0.11	1.1 ± 0.08	−0.164	0.874
DRD2	1.2 ± 0.39	1.1 ± 0.19	0.223	0.829
DRD3	1.0 ± 0.04	1.1 ± 0.10	−1.137	0.289
DRD4	1.0 ± 0.06	1.2 ± 0.28	−0.825	0.436
DRD5	1.0 ± 0.12	0.9 ± 0.03	0.794	0.453
Binding sites in IL (nCi/g T.E.)				
D1R	3.8 ± 0.38	3.0 ± 0.44	1.394	0.206
D2R	5.0 ± 0.26	5.5 ± 0.20	−0.364	0.734
D1R/D2R	0.77 ± 0.11	0.56 ± 0.14	1.218	0.278
Binding sites in PL (nCi/g T.E.)				
D1R	3.0 ± 0.17	2.7 ± 0.35	0.855	0.421
D2R	4.7 ± 0.44	4.9 ± 0.30	−1.186	0.280
D1R/D2R	0.74 ± 0.17	0.62 ± 0.08	0.705	0.531

Data are mean ± sem. *n* = 3–6 per strain. Analysed by Student’s *t*-test.

*BL6* C57BL/6J mouse, *D1R* dopamine D1 receptor, *D2R* dopamine D2R, *DRD1-5* dopamine receptor genes 1-5, *IL* infralimbic cortex, *PL* prelimbic cortex, *S1* 129S1/SvImJ mouse, *TE* tissue equivalent.

## Discussion

A major aim of the current study was to determine whether the mPFC serves as a key neural locus for the extinction-rescuing effects of L-DOPA in a model of deficient extinction (the S1 inbred mouse strain [44, 63]). Our findings show that the systemic delivery of L-DOPA augments extinction-related DA levels in mPFC and generates an extinction memory in S1 mice that persists for one, but not ten days. Conversely, direct microinfusion of DA (but not D1-like or D2 receptor agonists) into IL produced a robust and long-lasting rescue of extinction. Finally, we found evidence of abnormal DA-mediated neuronal responses in IL of S1 mice, suggesting a possible mechanism to account for the lack of long-term extinction-rescuing effects of L-DOPA.

Stimulation of the vmPFC has been proposed as a therapeutic approach to facilitate fear extinction in individuals with PTSD [80], with promising preliminary results [81]. Cross-species translational studies have shown that a single dose of L-DOPA produces long-term facilitation of fear extinction and associated increases in the activity of the vmPFC/IL in extinction-competent human subjects and rodents [12, 13]. Here, replicating and extending previous data in extinction-competent rats ([49, 50], but see [82]), we found extinction training was associated with increased extracellular mPFC DA availability in S1 mice. Specifically, DA levels were elevated during the first five minutes of training and peaked in the second half of the training session, indicating these effects were unrelated to handling or the novelty of the extinction context [83]. These behaviour-related mPFC DA levels were augmented by systemic L-DOPA administration, in line with a previous finding in rats tested in a cocaine-seeking procedure [55]. These data are notable because although the vmPFC/IL is strongly implicated in fear extinction, prior work had not determined a causal contribution of DA neurotransmission in this brain region to fear extinction (for review see [9, 11]).

Of further importance in this regard, systemic L-DOPA rescued extinction in S1 mice that was evident one day (see also [20]) after training. However, this effect was absent ten days post-training (see also [20]). These findings in mice agree well with recent data showing reduced efficacy of systemic L-DOPA in facilitating fear extinction in female patients with PTSD [19], relative to healthy individuals [12–14]. In contrast to the time-limited effects of systemic L-DOPA, we demonstrate that infusing DA into IL is in and of itself sufficient to generate an extinction memory in S1 mice. While the mechanisms underlying this effect remain to be elucidated, we posit that elevated DA levels enhanced IL neuronal excitability to promote extinction [30, 39, 40, 84, 85]. These findings also suggest that, unlike direct DA delivery to IL, systemic L-DOPA may not increase DA levels in this region beyond a threshold level necessary to generate a persistent extinction memory in otherwise compromised subjects. In an attempt to mitigate this putative DA deficit, we gave S1 mice L-DOPA both pre and post extinction training (and even in combination with benserazide, as common prescription in clinical practice, in order to reduce its peripheral degradation), but this treatment regimen was also unable to produce a persistent extinction rescue.

Another possibility is that reduced efficacy of L-DOPA is related to abnormal mPFC DA neurotransmission in extinction-comprised subjects. Indeed, there is an increasing number of reports that central DA markers, including the DA transporter, the DA degrading enzyme COMT, D1 and D2 receptors, are abnormal in patients with an anxiety disorder or PTSD [86–96]. In IL of S1 mice, abnormal DA signalling could arise from deficits in DA release from terminals of VTA and/or locus coeruleus [55] or in synaptic signalling at IL neurons. We were able to demonstrate that, relative to BL6 mice, S1 mice exhibited attenuated electrophysiological mPFC neuronal responses to DA and blunted IL neuron molecular signalling responses (notably including *Arc* [64, 65]) to L-DOPA and DA receptor agonists.

These analyses also showed that L-DOPA modified the activity of a subset of CaMKIIα-negative neurons (likely primarily interneurons) recruited during extinction training. Since interneurons can either synchronize the output of excitatory pyramidal cells [97, 98], increase cortical output via disinhibition [99] or broadly reduce mPFC neuronal output [100, 101], the consequences of extinction-related interneuron activation are difficult to predict. D1 receptors are expressed on VIP-positive interneurons which enhance action potential firing by influencing excitatory and/or disinhibitory microcircuits in deep and superficial layers of the mPFC, respectively [102]. Thus, D1 receptor dysfunction in S1 mice could result in a hypoactive IL ([44], but see [103]) in response to extracellular DA released during fear extinction training. In this context, social anxiety disorder and obsessive-compulsive disorder are associated with decreased D1 receptor binding [92] and increased D2 receptor in cortical subregions [91], with the latter being lower after successful psychotherapy [104].

Phasic DA release of the type that likely occurs during extinction favours activity at D1 over D2 receptors [105], whereas high levels of mPFC DA increases activation of D2 receptors expressed on pyramidal mPFC neurons [106], which in turn has been shown to increase response-related activity of layer 5 neurons in the PFC [107] via activation of NMDA receptors [108, 109] and recruitment of a G_s_ protein-coupled signalling pathways. Hence, elevated DA produced by DA or L-DOPA might generate a high neuronal activity DA state that shifts mPFC network responses from D1 receptor dominated mode of transmission to one that recruits other DA receptors [110, 111], potentially promoting extinction-enabling processes such as arousal and flexible behavioural control [112].

While additional work is needed to assess this hypothesis, local agonist activation of mPFC D1-like or D2 receptors did not affect within-session extinction in S1 mice (current study). Together with our finding of D1 receptor dysfunction in S1 mice, we posit that, despite triggering some level of downstream signalling following D1 receptor stimulation, the effect of D1 or D2 agonism is insufficient to rescue fear extinction in S1 mice or, alternatively, that IL D1 receptors are not involved in fear extinction acquisition. This latter possibility is supported by previous studies in extinction-competent subjects [26–29]. Earlier studies have also found that D2 receptor agonism impairs fear extinction, while D2 blockade facilitates extinction [28, 113], therefore it is unlikely that selectively activating D2 receptors would improve extinction in S1 mice. Instead, these data suggest that other DA receptors, including the D3 and D4 subtypes, or combined actions at multiple DA receptors, underlie the extinction-promoting effects of DA and L-DOPA in S1 mice. Indeed, we have previously found that deficient fear extinction in S1 mice is associated with upregulated D3, D4 and D5 receptor expression in mPFC [20]. Future studies examining the functional role of these receptor subtypes would be valuable in deciphering the cause of L-DOPA’s time-limited effects on fear extinction.

In sum, the current findings demonstrate that delivering DA into IL caused long-lasting rescue of deficient fear extinction in a clinically relevant model organism whereas, despite a pronounced rise in mPFC DA, systemic administration of L-DOPA produced shorter-lasting extinction-rescuing effects. Our results suggest that abnormal neuronal and intracellular signalling responses to DA in IL could account for the reduced efficacy of L-DOPA in the extinction-deficient mouse model employed. By extension, these findings support the idea that the use of systemic L-DOPA as a pharmacological adjunct to exposure-based therapy could be particularly efficacious in individuals with impaired fear extinction, but may be of limited efficacy in patients with severely impaired extinction which relies on similar or even same neurobiological abberations as observed in our mouse model.

### Supplementary information

Supplementary Text

## Data Availability

The corresponding author will make available the relevant data upon request. Additional data are provided in supplementary tables of this manuscript.

## Materials availability

Additional information on materials will be made available from the corresponding author upon request.
