# Supplementary material for: Fear extinction rescuing effects of dopamine and L-DOPA in the ventromedial prefrontal cortex
Source: Transl Psychiatry. 2024 Jan 8;14:11. doi: 10.1038/s41398-023-02708-8 (PMC10774374; doi:10.1038/s41398-023-02708-8)

Fear extinction rescuing effects of dopamine and L-DOPA in the ventromedial prefrontal cortex

Simone B. Sartori^1^, Thomas M.V. Keil^1^, Kai K. Kummer^2^, Conor Murphy^1^, Ozge Gunduz-Cinar^3^, Michaela Kress^2^, Karl Ebner^1^, Andrew Holmes^3^and Nicolas Singewald^1*^

^1^Department of Pharmacology and Toxicology, Institute of Pharmacy and Center for Molecular Biosciences Innsbruck, University of Innsbruck, Innsbruck, Austria

^2^Institute of Physiology, Department of Physiology and Medical Physics, Medical University of Innsbruck, Innsbruck, Austria

^3^Laboratory of Behavioral and Genomic Neuroscience, NIH/NIAAA, Rockville, MD, USA

## *Corresponding author:

Nicolas Singewald

Institute of Pharmacy, Department of Pharmacology and Toxicology

Center for Molecular Biosciences Innsbruck (CMBI)

Leopold Franzens University Innsbruck

Innrain 80-82

A-6020 Innsbruck, Austria

Tel: +43-512-507-58802

Fax: +43-512-507-58889

E-mail: [nicolas.singewald@uibk.ac.at](mailto:nicolas.singewald@uibk.ac.at)

Supplementary Text

Figure S1

Tables S1

### Supplementary Text

## Animals

Male, adult S1 and BL6 mice, either derived from an in-house breeding colony or purchased from the Jackson Laboratory, were group-housed in individually ventilated cages with food and water available ad libitum under standard vivarium conditions (22 ± 2°C temperature, 50-60% humidity, 12 h:12 h light/dark cycle with lights on at 7:00 a.m.) in an SPF-unit at the Center for Chemistry and Biomedicine, Innsbruck, Austria. Sample sizes for obtaining statistical significance were estimated according to the ARRIVE guidelines and based on our previous work involving comparable experiments. Mice were allocated to different experimental groups by a randomized block design, with each block consisting different strains and/or treatment. Mice within a block were assigned based on various home cages and body weight reaching comparable mean body weights per experimental group. Furthermore, during the experiment, strains and/or treatments were randomly alternated in sequence and two different test chambers.

## Stereotaxic surgery

Commercially available guide cannulae were unilaterally or bilaterally chronically implanted into the brains of anaesthetized (5 mg/kg xylazine, 80 mg/kg ketamine, i.p.) and unresponsive mice under semi-sterile conditions using a stereotaxic frame as previously described ^1^. The tips of the guide cannulae were positioned above the left and/or right IL using optimised coordinates relative to bregma ^2^: AP (anteroposterior) +1.2 mm, ML (mediolateral) +0.5 mm, DV (dorsoventral) -1.7 mm for S1 mice and AP +1.7 mm, ML +0.5 mm, DV -1.7 mm for BL6 mice. The guide cannulae were closed with dummy stylets. After surgery mice were single housed and received buprenorphine (0.5 mg/kg s.c.) and meloxicam (1.0 mg/kg p.o. via the drinking water) for analgesic care for up to three days. They were allowed to recover for 5‑7 days meanwhile they were habituated to the experimenter and experimental procedures. At the end of each experiment mice were euthanised with an overdose of thiopental and brains were removed. For histological verification of probe placement, frozen coronal sections (40 µm) were prepared and stained with cresyl violet. Only animals with correct probe placement were included in the analysis **(Figure 1B, 2A, 2C, 2E, 3B)**.

## Extinction of cued conditioned fear

Unless otherwise stated, fear conditioning and fear extinction training were performed in a fully automated fear-conditioning system (TSE, Technical & Scientific Equipment GmbH, Bad Homburg, Germany) following an ABBB design as previously described ^1,3^. Mice were fear conditioned by three pairings of a tone cue (75 dB, 30 s; CS) with a co-terminating mild, scrambled foot shock (0.6mA, 2 s; US) every 2 minutes in a clean (using water), brightly lit (300 lux) standard conditioning chamber (context A; 25 x 25 x 35 cm Perspex arena with a metal grid floor). On the next day, animals were moved to the extinction context B (25 x 25 x 35 cm dark arena with black walls and a solid grey floor illuminated with red light at 10 lux) that differed from context A in visual, olfactory (cleaned with 100 % ethanol) and spatial signals where they were exposed to 16 CS separated by 5 s intervals. In case, L‑DOPA or vehicle was administered i.p. one hour before extinction training. Tests for extinction retrieval on experimental day 3 and spontaneous recovery of fear on experimental day 13 were performed by the presentation of two CS with a 5 s interval in the extinction context B ^1^. In all sessions, animals were allowed to habituate to the test context for two minutes prior to the first CS and remained there for another two minutes after the final CS before being returned to their home cages. The percentage of CS-induced freezing behaviour, i.e. the absence of all non-respiratory movements ^4,5^, was analysed by an experienced observer blinded to treatment groups and averaged in two CS bins.

## In vivo microdialysis measurements of extracellular mPFC DA

On experimental day 1, mice were fear conditioned in context A (conditioning chambers, 26 x 30 x 32 cm, Coulbourn Instruments, Allentown, USA) and moved back to their home cages. A few hours later a microdialysis probe (MAB 4.15.1 PES, Microbiotech, Sweden) was carefully inserted into the guide cannula (MAB 4.15 IC, Microbiotech, Sweden, OD 0.48 mm, ID 0.35 mm) located above the right IL replacing the dummy cannula. Animals were gently transferred to their novel specific plexiglas microdialysis home cages and allowed to habituate overnight. The microdialysis probe with a PES membrane of 1 mm length reaching into the IL was connected to a microdialysis syringe pump (CMA, Stockholm, Sweden) via polyethylene tubing and a swivel-tether system suspended from a counterbalance lever arm mounted on the cage wall. Microdialysis probes were constantly superfused with sterile artificial cerebrospinal fluid (aCSF; consisting of in mM: 140 NaCl, 3.0 KCl, 1.2 CaCl_2_, 1.0 MgCl_2_, 1.0 Na_2_HPO_4_, pH 7.4) at a perfusion rate of 0.5 µl/min overnight that was increased to 1.0 µl/min in the morning of the experimental day. After an equilibration period of one hour microdialysis fractions were continuously collected every 10 minutes into ice-cooled microtubes containing an antioxidative mixture (in mM: 0.27 Na_2_EDTA, 100 acetic acid, 0.0125 ascorbic acid) vortexed and stored at -80 °C until further analysis. On day 2, the first ten microdialysis fractions were sampled in the home cage as baseline values. Then animals were moved to context B (26 x 20 x 13 cm standard cage illuminated by dim red light, swiped with ethanol) and after one hour 16 CS were presented for fear extinction training where two 5-minute microdialysis fractions were sampled. Thereafter, animals were returned back to their home cages and another ten 10‑minute microdialysates were harvested. Finally, microdialysis probes were perfused with high (100 mM) KCl containing aCSF in order to elicit local depolarization for testing functionality of the microdialysis systems. At any times the dead volumes by the inlet and outlet system were considered by a temporal delay in fraction collection. At the end of the experiment the animals were euthanised and brains removed and snap-frozen for histological verification of the microdialysis probe. Two animals were excluded due to misplaced probe from the analysis.

## Quantification of dopamine concentrations in microdialysates

DA concentrations in microdialysates were analysed in 5 µl fractions by high-performance liquid chromatography (HPLC) with electrochemical detection. The HPLC system consisted of a system controller (CBM-20A, Shimadzu, Kyoto Japan), degassing unit (DGU-20A3R, Shimadzu, Kyoto Japan) and a micro HPLC pump (LC-20ADXR, Shimadzu, Kyoto Japan) which was operated at a flow rate of 50 µl/min. Samples were injected via a SIL-20ACHT autosampler (Shimadzu, Kyoto, Japan) and separated on a C18 reversed-phase column (Inertsil ODS-3; 50 mm x 1.0 mm ID; 3 µm particle size; GL Sciences Inc, Japan). The HPLC system was coupled to a DECADE II electrochemical detector equipped with a SenCell amperometric flow cell (2 mm glassy carbon working electrode, Ag/AgCl reference electrode, Antec, Zoeterwoude, The Netherlands). The column and detector cell were maintained at 35°C by a column oven as part of the electrochemical detector. The applied potential was set to + 460 mV vs. reference electrode and was adjusted to a detection range of 100 pA/V with a filter frequency setting of 0.01 Hz. The mobile phase consisted of 93 % v/v buffered aqueous solution [in mM: 50 phosphoric acid, 50 citric acid, 2.36 octane-sulfonic acid, 0.1 Na_2_EDTA, pH adjusted to 5.6 with 50 % (w/w) NaOH] and 7 % (v/v) methanol that was filtered through a 0.22 µm cellulose acetate membrane before use. Under these conditions the detection limit for DA was 0.15 fmol/5 µl sample (on column) with a signal‑to‑noise ratio of at least 2. Calibration curves were constructed by matrix matched external standards (in the range of 50 pM to 1 nM of DA injected) and linear correlation coefficients higher than 0.999. The chromatograms were processed with LabSolution CS (version 5; Shimadzu, Japan). The area-under-the-curve (AUC) was determined and converted into concentration units using standard calibration equation. DA concentrations were expressed as absolute values (fmol/5 µl) in 10 minutes and 5 minutes microdialysates, respectively.

## intra-IL drug administration

Dummy stylets were removed and bilateral microinjection cannulae systems (PlasticsOne, USA) were inserted into the brains of fear conditioned animals via the guide cannluae reaching 1 mm beyond into the IL **(Figure 2A,C,E)**. 20 minutes prior fear extinction training agonist solutions or vehicle were infused at the given dose that was selected based on the binding affinities to the corresponding receptor and/or previous comparable studies in a volume of 0.2 µL at a rate of 0.1 µL/min using Hamilton syringes and microinjection pumps (Stoelting, USA) and left in place for another minute before being removed. A mixture of the pan-α (phentolamine, 20 µg/side) and pan-β (timolol, 20 µg/side) adrenoreceptor blockers was infused 10 minutes prior to local application of DA in order to allow a full occupation of adrenoceptors in the IL. DA (40 μg/side), the D1-like agonist SKF-81297 (0.1 μg/side), the D2 receptor agonist suminarole (10 μg/side), timolol and phentolamine were dissolved in aCSF. Control animals received the same volume of vehicle via this application route.

## Multi-electrode array (MEA) mPFC neuronal recordings

Acute coronal brain slices, containing the mPFC, were prepared from naïve S1 and BL6 mice as described previously ^6^. Briefly, animals were deeply anesthetized with isoflurane and their brains were rapidly removed and immersed in ice-cold oxygenated (95% O_2_, 5% CO_2_) aCSF (in mM: 125 NaCl, 25 NaHCO_3_, 25 D-glucose, 2.5 KCl, 1.25 NaH_2_PO_4_, 2 NaCl_2_ and 1 MgCl_2_, pH 7.4). Using a microtome (Leica Microsystems), coronal slices (300 µm) of the rostral mPFC (encompassing the IL, PL and Cg subdivisions) were cut in ice-cold aCSF and incubated for 10‑15 minutes in 32‑34°C warm oxygenated protective aCSF (in mM: 110 N-methyl-D-glucamine, 110 HCl, 2.5 KCl, 1.2 NaH_2_PO_4_, 25 NaHCO_3_, 25 D-glucose, 10 MgSO_4_, 0.5 CaCl_2,_ pH 7.4) and transferred to standard aCSF at room temperature for at least one hour prior electrophysiological recordings. Brain slices were fixed with a platinum-framed nylon grid onto planar MEA chips (120MEA100/30iR-ITO, Multi Channel Systems; **Figure 4A)** and superfused with oxygenated aCSF at 32‑34°C. Using a MEA2100 recording system (Multi Channel Systems) mounted onto an inverted Leica DMi1 microscope (Leica Microsystems), spontaneous action potential discharge activity was recorded at a sampling rate of 25 kHz during the first five minutes when DA receptor agonists were added to the bath for five minutes. The position of the brain slice on the electrode field was photographed using a Leica MC120 HD digital microscope camera (Leica Microsystems), for assignment of the individual electrodes to the respective brain regions **(Figure 4A)**.

## Quantitative polymerase chain reaction (qPCR)

Total RNA was isolated from homogenized tissue punches containing both IL and PL subregions which were derived from snap-frozen brains of naïve S1 and BL6 by the use of the TRI Reagent method (Sigma-Aldrich) according to the manufacturer’s guidelines. Only samples with a nanodrop quality measure of 260:280 > 1.70 were included in qPCR analysis. For the profiling of mRNA transcript levels, 500 ng of total RNA was reverse transcribed using qScript^TM^ cDNA SuperMix (Quanta Biosciences, USA) according to manufacturer’s protocol and obtained cDNA was diluted 1:15 with RT-PCR grade water (Ambion). qPCR was carried out using FAST SYBR master mix (Applied Biosystems) on a 7500 FAST system (Applied Biosystems) with exon specific primers (Eurofins MWG, Ebersberg, Germany) for Drd1a, (forward: 5’‑GTCTCCCAGATCGGGCATTT‑3’; reverse: 5’‑CCCCAAGGAATGCATAGG CT‑3’), Drd2 (forward: 5’‑CCAGTGAACAGGCGGAGAAT‑3’; reverse: 5’‑TAGACCGTGGTGGGATGGAT‑3’), Drd3, (forward: 5’‑CTAGTGGTGAGCCTGGCTGT‑3’; reverse: 5’‑TGAAATTCCAGACTCCACCTG‑3’), Drd4 (forward: 5’‑ATCGTGAGCCTGGCTGCT‑3’; reverse: 5’‑GAGCCCCCGTCTCTGTGA‑3’), Drd5 (forward: 5’‑GGAGCTTGACTGTGAGGAGG‑3’; reverse: 5’‑GCCCATGAGGGTGTTTCTAA‑3’) and GAPDH (forward: 5’‑ACCCAGAAGACTGTGGATGG‑3’; reverse: 5’‑CACATTGGGGGTAGGAACAC‑3’). All qPCR reactions were carried out in triplicates. Expression levels of the genes of interest were determined relative to GAPDH and transformed using the 2-ΔCt method.

## Quantitative D1-like and D2-like receptor autoradiography

Coronal sections (20 μm) were prepared from snap-frozen brains derived from naïve S1 and BL6 mice, mounted onto slides and stored at ‑80°C. For quantitative receptor autoradiography, mPFC-matched brain sections were warmed to room temperature and pre-treated with assay buffer (in mM: 50 TRIS, 120 NaCl, 5 KCl, 2 CaCl_2_ x 2H_2_0, 1 MgCl_2_ x 6 H_2_0; pH=7.4) for one hour at room temperature. Subsequently, sections were incubated in assay buffer containing [^3^H]‑SCH-23390 (1nM; PerkinElmer) in the presence of the 5‑HT_2A_ receptor antagonist ketanserin (100 nM) for visualizing D1-like receptors and [^3^H]‑nemonapride (1nM; PerkinElmer) in the presence of the β‑adrenoceptor antagonist pindolol (0.1 µM) and the σ_1/2_ receptor antagonist DTG (0.5 µM) for visualizing D2-like receptors, respectively, for one hour at room temperature according to previous protocols ^7^. Non-specific binding of the [^3^H]‑labeled radioligands was determined by adding an excess (10 µM) of non-radioactive D1 receptor antagonist R(+)SCH23390 or D2/3R antagonist S(-)sulpiride. At the end of incubation, the sections were washed twice in ice-cold assay buffer, rinsed in ice-cold deionized water and air-dried overnight. The slide-mounted sections were exposed to a ^3^H‑sensitive phosphor-screen (GE Healthcare, UK) together with autoradiographic [^3^H]-microscales (Amersham Biosciences). After nine days of exposure, images were generated by a phosphor-imager (Typhoon FLA 7000, GE Healthcare, Sweden). Quantitative analysis of D1-like and D2-like receptor expression in IL and PL subregions, which were identified with reference to a mouse brain atlas ^2^, was performed in two sections per brain by an experimenter blinded to mouse strain using a computerized image analysis system (Image J, NIH, USA). Grey values were converted into optical densities and further into nCi/g tissue equivalent using [^3^H]-microscale standards with corrected activity levels for decay time from the time of purchase to present use.

## Arc fluorescence in situ hybridization (FISH) in mPFC neurons

FISH was performed on 12 µm frozen tissue sections mounted on gelatine-coated slides as previously described in detail ^8^. A customised, pre-labelled locked nucleic acid (LNA) probe with 5’ and 3’ fluorescein tags directed against the murine *Arc* mRNA (sequence: 5’ 6-FAM AATCTGGAGAGTGGCTGGGAT 3’ 6-FAM) was ordered from Exiqon (Exiqon, Denmark). Tissue sections were post-fixed with 4 % paraformaldehyde and acetylated in acetylation buffer, each followed by wash steps in diethyl‑pyrocarbonate-treated phosphate-buffered saline. Sections were pre-hybridized in hybridization buffer at 58 °C and subsequently incubated in hybridisation buffer containing the LNA probe (2.5 pM) at 58 °C for two hours. Post hybridization washes included three washes in 0.1 x saline sodium citrate (SSC) buffer at 60°C for 10 minutes followed by two 5 minute washes in 2 x SSC at room temperature. Subsequently, sections were treated with blocking buffer (3 % H_2_O_2_) for 30 minutes and incubated in blocking buffer containing primary mouse anti-Ca^2+^/calmodulin-dependent protein kinase II (CamKII) antibody (1:500, SantaCruz) overnight at 4°C. Sections were then washed and incubated in solutions containing both secondary anti-FITC (1:1000; Sigma) and donkey anti-rabbit (1:1000, Life Technologies) antibodies for 1 h at room temperature. Amplification of fluorescent signal was achieved by using the tyramide signal amplification (TSA) system (Perkin Elmer) according to the manufacturer’s instructions. Slides were coverslipped with the help of Prolong Gold mountant containing DAPI (Invitrogen) and imaged using a fluorescent Olympus BX51 microscope and the CellSense software (Olympus Corporation, Tokyo, Japan). The same exposure time was applied to all sections in order to acquire images across all mice in the green, red and blue channels. The expression of *Arc* mRNA was bilaterally quantified in a representative area of 0.02 mm² in each region of interest by a person blinded to experimental groups. The number of DAPI-positive and CamKII-positive cells expressing cytoplasmic, nuclear or cytoplasmic + nuclear *Arc* mRNA was determined. The number of *Arc* mRNA-positive cells in the different conditions and compartments were normalised to the number of DAPI-positive cells or cytosolic *Arc* mRNA expression of control group (vehicle treatment or BL6 mice) and expressed as relative value.

## IEG immunohistochemistry of pERK and pCREB in mPFC neurons

15 minutes or one hour after systemic administration of L‑DOPA (20 mg/kg) or SKF‑81297 (10 mg/kg) mice were euthanised with an overdose of thiopental and transcardially perfused with saline and 4 % paraformaldehyde solution. Brains were dissected, post-fixed with 4 % paraformaldehyde solution for two hours and stored in phosphate-buffered saline (0.2 M) at 4°C. Free-floating coronal brain sections (40 µm) of the mPFC were quenched with 1 % H_2_0_2_ in Tris-buffered saline (TBS; pH=7.4) for 30 minutes and, after three washes with TBS sections were treated with a TBS blocking solution containing 0.1 % Triton-X and 1 % bovine serum albumin for another 30 minutes. Sections were then incubated in primary rabbit anti-pERK (1:1000; Cell Signalling Technology, USA, #4370) or anti-pCREB (1:1000; Cell Signalling Technology, USA, #9198) antibodies for 48 h at room temperature. After three rinses with 0.1 % Triton-X-TBS, sections were incubated with a secondary biotinylated goat anti-rabbit antibody (1:500; Vectastain ABC kit, Vector Laboratory, USA) at room temperature for 24 hours followed by incubation in an avidin-biotin-complex solution (Vectastain ABC kit, Vector Laboratory, USA) for two hours and in 3,3'-diaminobenzidine solution for 10 minutes. A chromogenic reaction was initiated by adding 0.1 % H_2_0_2_ and stopped with Tris buffer when color changes were observed and no further darkening of the slices was expected. Washed sections were mounted on glass slides, dried overnight and coverslipped. Cells with brown, spherical reaction products were considered as pERK- and pCREB-positive, respectively, and quantified in the IL, PL, cingulate cortex and motor cortex. The localisation of cells from digitised images taken via a light microscope (Olympus BX-40; 10x) was aided with illustrations in a stereotaxic atlas ^2^. Quantification of pERK- and pCREB-positive cells was manually performed by a trained experimenter blinded to experimental groups with assistance by an imaging software (CellSense Dimension 1.5, Olympus Corporation, Tokyo, Japan). The number of pERK- and pCREB-positive cells in a representative region of 0.01 mm^2^ was quantified bilaterally in each brain area of interest and, where appropriate, in different layers.

## Statistical analysis

Data are expressed as mean ± sem. Statistical analysis of data was performed using Statistica 13 (StatSoft Europe) following the exclusion of outliers identified by Grubb’s test. Shapiro Wilk W test was applied for assessing a normal distribution of the data sets. Parametric data were analysed by an ANOVA with or without repeated measures followed by a post-hoc multiple comparisons test, when allowed, or an unpaired Student’s t-test (two-tailed). MEA recordings were analysed by GraphPad QuickCalcs (χ2 for 2x2 contingency tables; http://graphpad.com/quickcalcs/) or VassarStats (χ2 for 3x2 contingency tables; <http://vassarstats.net/>). The level of statistical significance was set to *p* < 0.05.

### References

1 Sartori SB, Maurer V, Murphy C, Schmuckermair C, Muigg P, Neumann ID *et al.* Combined Neuropeptide S and D-Cycloserine Augmentation Prevents the Return of Fear in Extinction-Impaired Rodents: Advantage of Dual versus Single Drug Approaches. *Int J Neuropsychopharmacol* 2016; **19**: 1–11.

2 Paxinos G, Franklin KBJ. *The Mouse Brain in Stereotaxic Coordinates*. 3rd ed. Academic Press: Amsterdam, 2007.

3 Whittle N, Maurer V, Murphy C, Rainer J, Bindreither D, Hauschild M *et al.* Enhancing dopaminergic signaling and histone acetylation promotes long-term rescue of deficient fear extinction. *Transl Psychiatry* 2016; **6**: e974.

4 Blanchard RJ, Blanchard DC. Passive and active reactions to fear-eliciting stimuli. *J Comp Physiol Psychol* 1969; **68**: 129–135.

5 Fanselow MS. Conditioned and unconditional components of post-shock freezing. *Pavlov J Biol Sci* 1980; **15**: 177–182.

6 Kummer KK, El Rawas R, Kress M, Saria A, Zernig G. Social interaction and cocaine conditioning in mice increase spontaneous spike frequency in the nucleus accumbens or septal nuclei as revealed by multielectrode array recordings. *Pharmacology* 2015; **95**: 42–49.

7 Delis F, Mitsacos A, Giompres P. Dopamine receptor and transporter levels are altered in the brain of Purkinje Cell Degeneration mutant mice. *Neuroscience* 2004; **125**: 255–268.

8 Silahtaroglu AN, Nolting D, Dyrskjøt L, Berezikov E, Møller M, Tommerup N *et al.* Detection of microRNAs in frozen tissue sections by fluorescence in situ hybridization using locked nucleic acid probes and tyramide signal amplification. *Nat Protoc* 2007; **2**: 2520–2528.

9 Orsini CA, Yan C, Maren S. Ensemble coding of context-dependent fear memory in the amygdala. *Front Behav Neurosci* 2013; **7**: 199.

10 Lee HJ, Haberman RP, Roquet RF, Monfils M-H. Extinction and Retrieval + Extinction of Conditioned Fear Differentially Activate Medial Prefrontal Cortex and Amygdala in Rats. *Front Behav Neurosci* 2015; **9**: 369.

11 Guzowski JF, McNaughton BL, Barnes CA, Worley PF. Environment-specific expression of the immediate-early gene Arc in hippocampal neuronal ensembles. *Nat Neurosci* 1999; **2**: 1120–1124.

### Figure legends

**Figure S1.** Compartmental *Arc* mRNA expression pattern in the mPFC of mice following fear extinction training. **(A)** Scheme showing the experimental procedure for measuring *Arc* mRNA expression after extinction training, via catFISH. Since *Arc* mRNA exhibits dynamic subcellular localisation within neurons depending on the time interval from a stimulus, it allows to determine neuronal activity at different timepoints. Accordingly, while the abundance of intranuclear *Arc* mRNA points towards neuronal activity within 2-10 minutes before scarification, thus, providing a proxy for late fear extinction, cytoplasmic *Arc* mRNA expression would indicate neuronal activation approximately 30 minutes prior death reflecting the early phase of fear extinction, and indiscriminate labelling in both regions would reflect persistent activity throughout training (see also ^9–11^). Therefore, extinction training was temporally extended involving 30 CS rather than 16 CS so that peaks for cytoplasmic and nuclear *Arc* mRNA expression correlated with early (first five minutes) and late (last five minutes) extinction phases. **(B)** High magnification of representative image depicting cytoplasmic (green staining around the blue nuclear DAPI counterstaining), nuclear (green dots inside the blue nuclear DAPI staining) or cytoplasmic+nuclear (green staining around and inside the blue nuclear DAPI counterstaining) *Arc* mRNA expression in CaMKIIα-positive (red cytoplasmic staining) and CaMKIIα-negative cells in IL. **(C-F)** Fear extinction training induced *Arc* mRNA in cytoplasmic and nuclear cellular compartments of neurons in IL and PL. The total number of neurons expressing *Arc* mRNA in the cytosol and/or nucleus of did not differ between vehicle (n = 7) and L‑DOPA-treated (n = 7) S1 mice in the infralimbic cortex [treatment x compartment: F(2,35) = 0.800, *p*= 0.457]; **C**] and the prelimbic cortex [treatment x compartment: F(2,35) = 0.897, *p*= 0.7417; **D**]. However, treatment significantly increased *Arc* mRNA in CaMKIIα-negative cells of IL [treatment: F(1,35) = 12.871, *p* = 0.001; treatment x compartment: F(2,35) = 1.62, *p*= 0.212], an effect which seemed to be mostly present in cells persistently activated throughout training (as indicated by cytosolic and nuclear *Arc* mRNA expression), and not in PL [treatment: F(1,36) = 1.95, *p* = 0.172; treatment x sub-cellular compartment: F(2,36) = 0.854, *p*= 0.434] of S1 mice following L-DOPA treatment. **(G,H)** The number of neurons expressing extinction-related *Arc* mRNA was reduced in IL [line: F(1,41) = 6.260, *p* = 0.016], but not PL [line: F(1,41) = 1.565, *p* = 0.218], of S1 (n = 9) as compared to BL6 (n = 7) mice. This effect was present throughout all extinction training phases as indicated by cytosolic and/or nuclear *Arc* mRNA expression in IL [sub-cellular compartment: F(2,42) = 6.432, *p* = 0.004; strain x sub-cellular compartment: F(2,42) = 0.789, *p*= 0.461]. Data are expressed relative to cytosolic *Arc* mRNA expression of vehicle treatment and BL6 controls, respectively, and presented as mean ± sem. Analysed by two-way ANOVA followed by a Fisher‘s LSD test. Abbreviations: A: context A; B; context B; C, cyt: cytosolic *Arc* mRNA expression; C+N, cyt+nuc: cytosolic and nuclear *Arc* mRNA expression; CS: conditioned stimulus; d: day; N, nuc: nuclear *Arc* mRNA expression.

**Table S1.** Cortical expression of pERK and pCREB in S1 and BL6 mice following systemic administration of dopaminergic drugs

|  | **L-DOPA** | | **SKF-81297** | | **strain x treatment** | |
| --- | --- | --- | --- | --- | --- | --- |
|  | **BL6** | **S1** | **BL6** | **S1** | **F** | ***p*** |
| ***Cingulate cortex*** |  |  |  |  |  |  |
| pERK (15min) | 27.9 ± 2.4 | 25.4 ± 6.5 | 46.5 ± 7.7 | 48.9 ± 8.6 | 0.131 | 0.723 |
| pERK (1h) | 33.1 ± 0.3 | 38.4 ± 2.5 | 26.0 ± 2.9 | 26.8 ± 2.6 | 0.616 | 0.442 |
| pCREB(1h) | 121 ± 4.5 | 132 ± 6.0 | 107 ± 17 | 110 ± 7.6 | 0.137 | 0.716 |
| ***Motor cortex 2*** |  |  |  |  |  |  |
| pERK (15min) | 19.8 ± 2.9 | 22.1 ± 10.6 | 38.0 ± 9.2 | 36.3 ± 8.4 | 0.062 | 0.808 |
| pERK (1h) | 18.4 ± 2.5 | 26.4 ± 3.0 | 14.6 ± 0.8 | 15.9 ± 1.6 | 1.753 | 0.200 |
| pCREB(1h) | 116 ± 4.2 | 117 ± 6.6 | 111 ± 8.0 | 100 ± 8.2 | 0.484 | 0.494 |

Number of pERK or pCREB-positive cells/0.03 mm^2^ are given. Data are mean ± sem. n = 4-6 per experimental group. Analysed by two-way ANOVA. Abbreviations: SKF-81297: D1 receptor agonist.


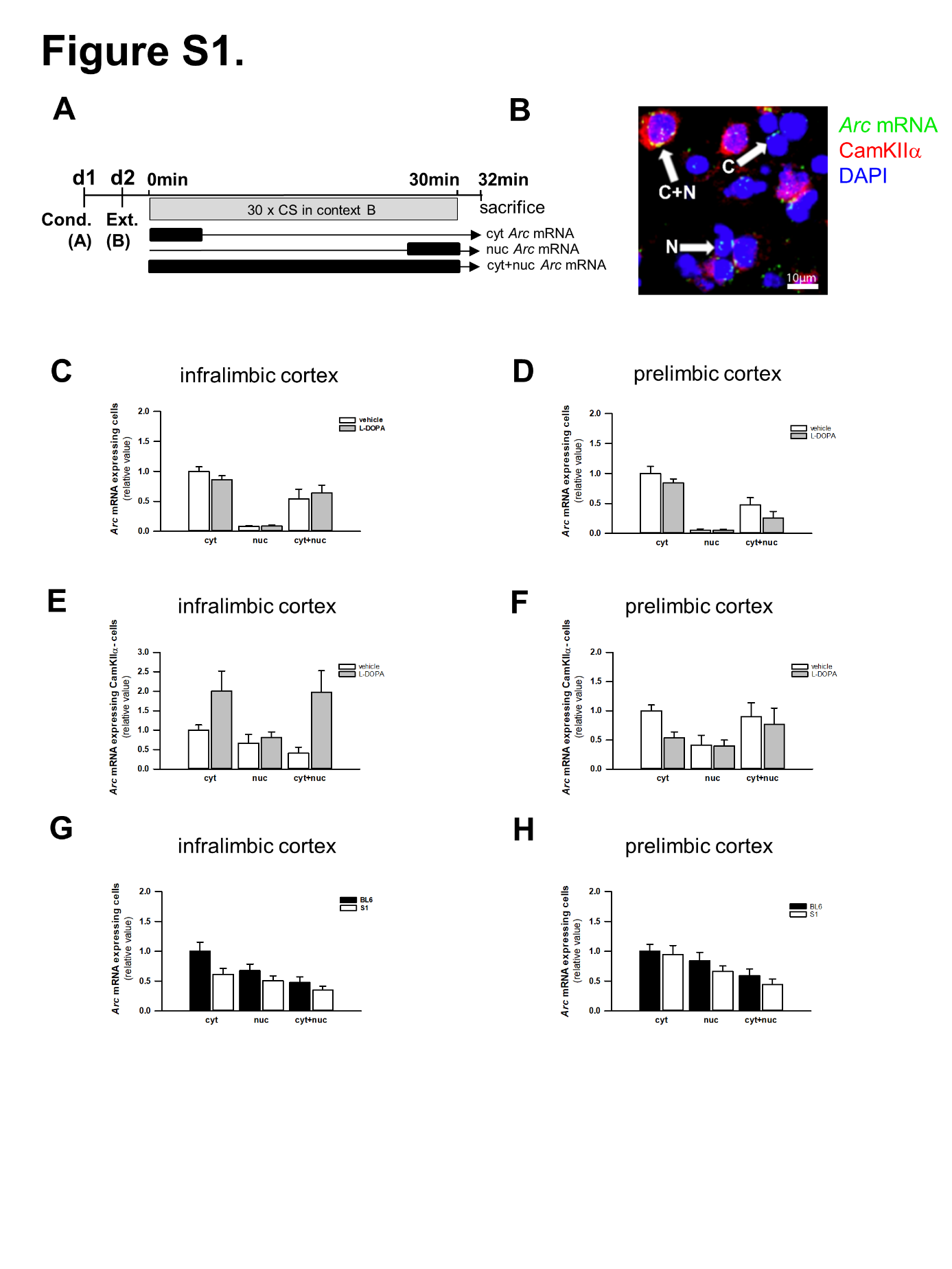

Supplement: Supplementary file 1 — Supplementary Text [file 41398_2023_2708_MOESM1_ESM.docx]
